# Optimization of Notification Criteria for Shiga Toxin–Producing *Escherichia coli* Surveillance, the Netherlands

**DOI:** 10.3201/eid2701.200339

**Published:** 2021-01

**Authors:** Ingrid H.M. Friesema, Sjoerd Kuiling, Zsofia Igloi, Eelco Franz

**Affiliations:** National Institute for Public Health and the Environment, Bilthoven, the Netherlands (I.H.M. Friesema, S. Kuiling, Z. Igloi, E. Franz);; European Program for Public Health Microbiology Training, Stockholm, Sweden (Z. Igloi)

**Keywords:** Shiga-Toxigenic Escherichia coli, epidemiology, public health, incidence, trends, E. coli, foodborne diseases, zoonoses, food safety, enteric infections, bacteria, the Netherlands

## Abstract

We describe the consequences of 2 major changes in notification criteria for Shiga toxin–producing *Escherichia coli* surveillance in the Netherlands. The change to reporting acute, more severe infections appears to be a good compromise between workload, redundancy, and public health relevance, provided isolates remain available for typing and sequencing.

Shiga toxin–producing *Escherichia coli* (STEC) is a zoonotic pathogen that causes illnesses ranging from mild diarrhea to hemolytic uremic syndrome (HUS) and death (*1*,*2*). Infection mainly occurs through consumption of contaminated food or contact with animals or manure. In most countries in Europe, STEC infections are notifiable at a national level. In 2017, the mean notification rate in Europe was 1.8 cases/100,000 population (*3*). Because STEC can cause severe disease and outbreaks, its notification is essential (*4*).

By combining epidemiologic case data with pathogen typing information, which has become increasingly genome-based in recent years, the Netherlands implemented STEC surveillance to follow trends in incidence and circulating types and detect and define outbreaks. STEC surveillance also provides data to inform public health actions to prevent and control further spread of the pathogen. 

The Netherlands started surveillance for STEC O157 in 1999. The introduction of PCR, especially PCR targeting the Shiga toxin–producing genes, facilitated diagnosis of all STEC and PCR was faster and more sensitive than the standard culture (*5*–*8*). A pilot study in the Netherlands during 2005–2006 showed the common presence of non-O157 STEC infections (*9*); subsequently, STEC O157 surveillance was extended to all STEC in July 2007. The extension caused an overload of reports at the regional public health service, with the result that case-level information about the disease and its course was missed. Furthermore, available information suggested that most reports were from cases with mild long-term symptoms. In July 2016, the notification criteria were narrowed to target acute, more severe STEC infections. We reviewed the effects of changes in notification criteria on STEC surveillance in the Netherlands.

## The Study

In the Netherlands, laboratories and medical doctors must report laboratory confirmed STEC cases to the regional public health service. The public health service subsequently reports cases to the National Institute for Public Health and the Environment (RIVM). Information gathered includes sex, birth year, date of illness onset, symptoms, hospitalization, and death. From its inception in January 1999, STEC surveillance in the Netherlands has been enhanced by laboratory surveillance in which laboratories are requested to voluntarily send STEC isolates to RIVM for further typing.

When STEC notification began in 1999, there were 2 criteria: 1) notification for every person with diarrhea, stomachache, or both; STEC O157 confirmed via a culture; or demonstration of Shiga toxin (Stx) or Shiga toxin genes (*stx*) in feces or bowel contents; and 2) notification for every person with HUS or with STEC O157 confirmed via a specific antibody response for STEC O157, a culture, or demonstration of Stx or *stx1* or *stx2* genes in feces or bowel contents. In July 2007, notification criteria shifted from notification for STEC O157 to notification of all STEC serotypes, using the same 2 criteria. Then, in July 2016, the first criterium was changed to notification of every person with diarrhea, blood in stool, vomiting or any combination, and <21 days between date of symptom onset and date of fecal sample taken in combination with STEC confirmation via a culture, demonstration of the combination of *stx1* gene with the *eae* or *escV* gene, or demonstration of *stx2* gene. 

During 1999–2019, a total of 8,307 STEC infections, including 230 HUS cases, were reported (Figure). After the change in notification criteria in 2007, we noted a 20-fold increase in reported STEC cases (Figure). Comparable increases were seen in the United States and other countries in Europe after introduction of methods for detecting all STEC serotypes (*10*–*13*). The 2016 criteria change for notification halved the number of reported cases. Introduction of molecular diagnostics also led to omission of culturing strains for further typing for most cases, which is especially apparent since 2018.

**Figure:**
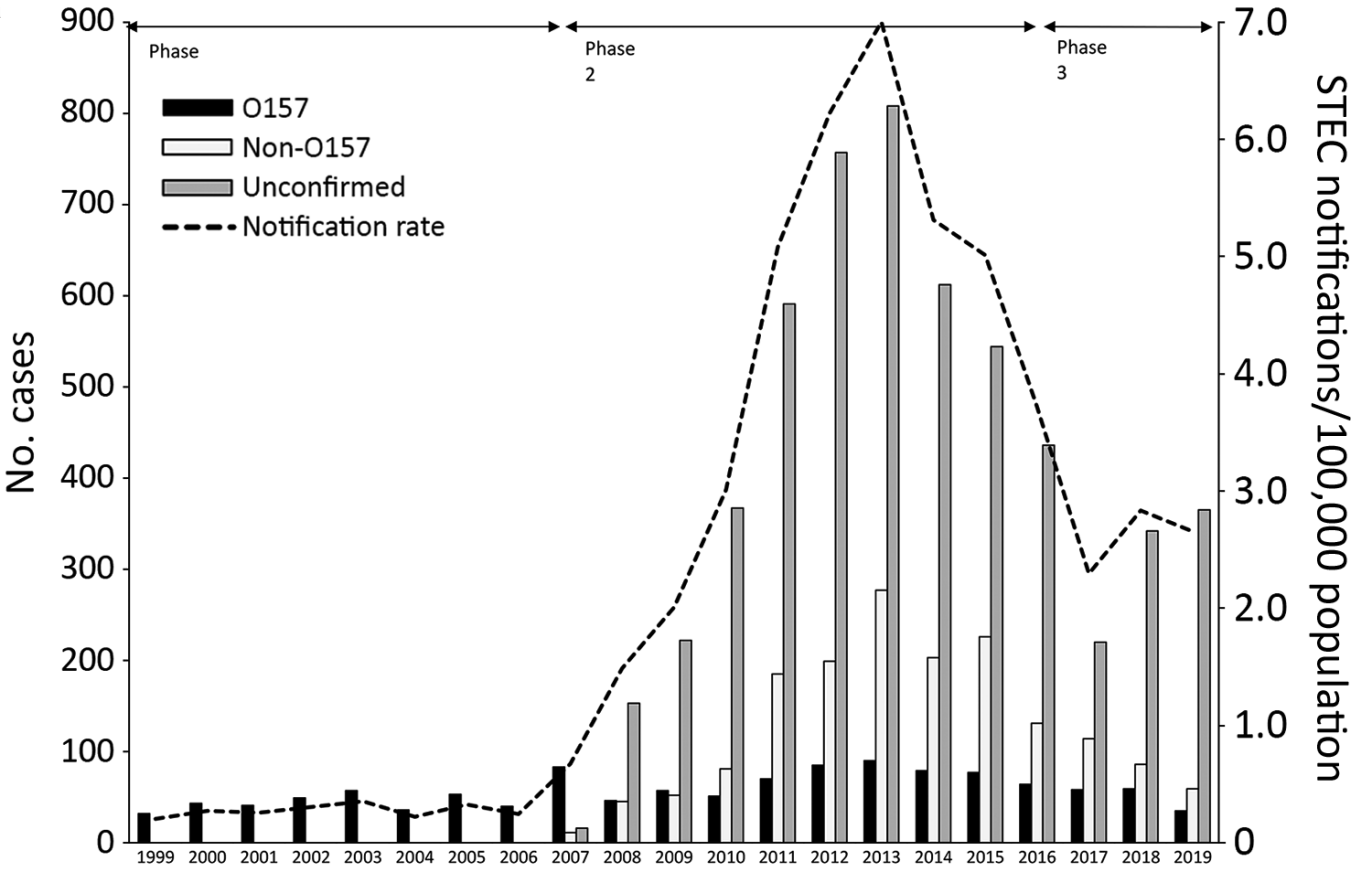
Number of reported Shiga toxin–producing *Escherichia coli* (STEC) infections per STEC group, the Netherlands, 1999–2019. In total, 8,307 infections were reported. For unconfirmed cases, no isolate was sent in for typing or isolates sent to the National Institute for Health and the Environment could not be confirmed or typed. During phase 1, 1999–June 2007, notification criteria were 1) notification for every person with diarrhea, stomachache, or both; STEC O157 confirmed via a culture; or demonstration of Shiga toxin (Stx) or Shiga toxin genes (*stx*) in feces; and 2) notification for every person with HUS; STEC O157 confirmed via a specific antibody response for STEC O157, a culture, or demonstration of Stx or *stx1* or *stx2* genes in feces. During phase 2, July 2007–June 2016, notification criteria shifted from notification for STEC O157 to notification of all STEC serotypes using the same 2 criteria. During phase 3, July 2016–December 2019, the first criterium was changed to notification of every person with diarrhea, blood in stool, vomiting or any combination, and <21 days between date of symptom onset and date of fecal sample taken in combination with STEC confirmation via a culture, demonstration of the combination of *stx1* gene with the *eae* or *escV* gene, or demonstration of *stx2* gene.

STEC O157 is the only serotype for which the Netherlands has 21 years of available data on infections. The number of cases per year varied from 32 in 1999 to 90 in 2013. The highest annual numbers were seen in 2007 (83 cases, 41 of which were part of a national outbreak) and during 2011–2015 (70–90 cases/year). In other years, 32–64 cases were notified. The proportion of HUS cases was highest (12%–26%) during the early years of surveillance, 1999–2004; since 2005, HUS incidence varies from 2%–11% per year. The median number of HUS cases were 12.5 (range 5–23) cases during 2008–2015 and 21 (range 12–22) cases during 2017–2019. During July 2016–December 2019, median age among HUS cases was 29 years compared with median age of 18 years during 1999–2007 and 22 years during July 2007–June 2016. Hospitalization rates for STEC remained stable, fluctuating from 29% to 54% per year. 

In the Netherlands, STEC O26 is the most prevalent and severe non-O157 STEC serotype. Among 37 HUS cases reported to be caused by a non-O157 STEC, 20 (54%) were diagnosed STEC O26 infections. In addition, STEC O26 prevalence increased from 12% during July 2007–June 2016 to 27% during July 2016–December 2019 (Table 1). STEC O103 also moved from fourth most prevalent to second most prevalent after the last change in notification criteria; whereas STEC O91 dropped out of the top 5 most prevalent serotypes. Since July 2016, gene profiles *stx1* + *hly* and *stx1* + *stx2* + *hly* were seen less often in non-O157 STEC, but profiles *stx1* + *eae* + *hly* and *stx2* + *eae* + *hly* became more common (Table 2).

**Table 1:** Most prevalent serotypes in non-O157 Shiga toxin–producing *Escherichia coli*, the Netherlands, July 2007–June 2016 and July 2016–December 2019

Most prevalent	July 2007–June 2016, n = 1,370			July 2016–December 2019, n = 299	
	Serotype	No. (%)		Serotype	No. (%)
1	O26	171 (12)		O26	80 (27)
2	O91	137 (10)		O103	31 (10)
3	O146	89 (6)		O63	18 (6)
4	O103	87 (6)		O146	16 (5)
5	O63	54 (6)		O145	13 (4)
*Non-O157 includes O nontypeable serotypes. Because O nontypeable is a diffuse group, we left it out of the list of most prevalent serotypes. However, during July 2007–June 2016, 118/1,370 (9%) serotypes were O nontypeable; during July 2016–December 2019, 11/299 (4%) were O nontypeable.					

**Table 2:** Most prevalent genes in non-O157 Shiga toxin–producing *Escherichia coli*, the Netherlands, July 2007–June 2016 and July 2016–December 2019*

Most prevalent genes, no. (%)	July 2007–June 2016, n = 1,366					July 2016–December 2019, n = 292			
	*eae*	*hly*	*eae + hly*	None		*eae*	*hly*	*eae + hly*	None
*stx1*	18 (1)	170 (12)	287 (21)	130 (10)		3 (1)	7 (2)	95 (33)	10 (3)
*stx2*	4 (0.3)	99 (7)	82 (6)	173 (13)		3 (1)	18 (6)	53 (18)	35 (12)
*stx1* + *stx2*	0	158 (12)	52 (4)	30 (2)		1 (0.3)	12 (4)	21 (7)	6 (2)
*stx2f*	160 (12)	0	0	3 (0.2)		26 (9)	0	1 (0.3)	1 (0.3)
*eae, attaching and effacing gene; *hly*, hemolysin gene; *stx1*, Shiga toxin type 1 gene; *stx2*, Shiga toxin type 2 gene.									

## Conclusions

The introduction of PCR facilitated detection of all STEC in the Netherlands. However, STEC is a heterogeneous group and some serotypes are more prone to cause severe disease than others. Expanding surveillance to all STEC caused a 20-fold increase of reported cases, some of which were from cases with mild and long-term symptoms. Because PCR is faster, cheaper, and easier than culture, it might be requested more for rapid results in cases of less severe disease. Furthermore, many laboratories implemented reverse transcription PCR multiplex assays in which a specimen is tested for several diseases in a single run, instead of testing for 1 disease at a time. A study in Norway showed that in laboratories introducing a multiplex assay as standard detection method, the number of STEC reports, especially of low-virulence STEC, increased substantially compared with laboratories without this method (*13*). Introduction of a multiplex assay also lead to an increase in detection of concomitant infections.

Surveillance of STEC serotypes and virulence gene profiles remains vital and relevant. The confinement of the notification criteria to acute disease onset with >1 of 3 predefined symptoms increased the public health relevance of surveillance. The data did not show large effects of the criteria changes on STEC O157, which implies no noticeable effects on notifications of relatively severe disease within the surveillance. However, a new challenge emerged. Isolates are needed to provide information on confirmed STEC cases and circulating serotypes and are used for nationwide outbreak detection using whole-genome sequencing. In an era of increased molecular diagnostics, less culturing is performed by the regional laboratories, especially as serotype information is not relevant for treating patients. The current notification criteria in the Netherlands appear to be a good compromise between medical laboratory workload, redundancy of less public health-relevant cases, and the ability to carry out public health actions. However, we stress that national surveillance is threatened by reduced culturing and urge public health institutes and laboratories to coordinate to safeguard against loss of cultures in the future.
